# EMDR in Competition with Fate: A Case Study in a Chinese Woman with Multiple Traumas

**DOI:** 10.1155/2012/827187

**Published:** 2012-01-26

**Authors:** Maggie Wai-Ling Poon

**Affiliations:** Clinical Psychological Unit 3, Social Welfare Department, 14/F. Cornwall House, Taikoo Place, 979 King's Road, Quarry Bay, Hong Kong

## Abstract

This paper described the application of eye movement desensitization reprocessing (EMDR) for addressing the posttraumatic stress disorder (PTSD) symptoms in a Chinese woman who had experienced multiple traumas in her childhood. EMDR is an integrative therapeutic intervention that uses a standardized eight-phase approach to treatment. It is also a proven, effective, and efficient treatment for trauma. In this client with multiple traumas, the etiological event that lay the foundation of her dysfunctional responses was reprocessed first. The successful resolution of this event allowed the positive treatment effects to transfer to other traumatic events of a similar theme. This case also illustrates the importance of identifying a culturally appropriate positive cognition (PC) in contributing to the success of the treatment.

## 1. Introduction

Individuals exposed to multiple traumas may suffer from a variety of psychological problems, including posttraumatic stress symptoms (PTSD), anxiety, depression, affect dysregulation, dissociation, relational difficulty, and distorted self-perception (e.g., [1–3]). The severity of these complex conditions varies according to the age and developmental stage at which the trauma occurred. In general the younger the age the trauma occurs, the greater will be the impact [4].

The contemporary model for the psychological treatment of complex PTSD involves a 3-staged approach addressing to specific issues in different stages [2, 3, 5–7]. The first stage targets developing treatment alliance, teaching affect regulation skills, and fostering an internal sense of safety. The second stage focuses on reprocessing the traumatic memories. A variety of exposure-based psychotherapeutic approaches have been found to be effective for treating trauma [8]. Among them, eye movement desensitization and reprocessing (EMDR) is one of the evidence-based effective approaches [8–10] in the treatment of PTSD. The third phase of treatment aims at enhancing the daily living skills and fine-tuning of the self-regulatory skills which clients learn at the previous stages [6]. As noted by Curtosis [3, page 92], the treatment model for complex PTSD “is highly individualized depending on the client's needs and capabilities… recognizes that different healing patterns and prognoses are likely,” Thus the duration of the entire therapy may vary substantially from years or even decades in different clients [2].

EMDR focuses on reprocessing the significant memories that underpin the dysfunctional responses of the clients [11]. Significant memories refer to the unprocessed information of the crucial traumatic event that encapsulates the sensory and perceptual experiences inherent at the time of the event [12]. For effective treatment, the EMDR therapists should identify the earliest etiological event that lays the foundation of the pathology.

EMDR consists of eight-phases that address past traumatic events and present triggers of the symptoms [12]. The first two phases include history taking and preparation of the client. The third phase identifies the target traumatic memories, the negative cognition (NC) related to the event, and the positive cognition (PC) that the client prefers to associate with the event. The fourth phase consists of dual-attention stimulation in the form of repeated sets of eye movement, tones, or taps. The fifth, sixth, seventh, and eight phases constitute installation, resolving any residual somatic sensations, closure, and reevaluation. The case below described a Chinese woman who suffered from posttraumatic stress disorder resulting from experiencing multiple traumas in her childhood. 

## 2. Case Presentation

### 2.1. Presenting Symptoms

Mary (Mary is the pseudonym of the client), a 62-year-old Chinese lady with 5 years of education, consulted me for depressed mood, panic attack, and numbing response triggered by conflict with her adult daughter. Her emotional functioning was unstable with chronic feelings of fear, confusion, nightmares (dreamed of baby crying), forgetfulness, mind going blank, freezing response, and flashbacks. She was experiencing moderate impairment in her daily functioning. There were times that she discovered herself crossing a road without knowing why she got there, or she found herself got injured without feeling any pain. Prior to this consultation, she consulted a family physician who prescribed antidepressants to her.

Mary lived with her daughter and son-in-law. Her daily job was to take care of two school-aged grandchildren. She often had conflicts with her daughter over child discipline and daily household management. These conflicts set off her flashbacks of memories of traumatic situations (e.g., a ladder, an old village house, and blood on the floor). These intrusions caused her headache, stiffness of body, chest discomfort, ulcer pain, and nose bleeding. She fulfilled the criteria for complex PTSD [1, 3, 4].

### 2.2. From Symptoms to Targets

Mary reported to have encountered a number of stressful life events since early childhood. At the age of 6, she witnessed the accidental death of her brother; at 12, she saw her aunt kill her baby by throwing him from heights; at 16, she experienced her sister running away from home; at 28, she underwent an abortion because of contracting venereal disease from her husband; in her thirties, she was deserted by her spouse.

A detailed history taking revealed that the etiological event was the death of Mary's brother. This happened when Mary was 6 years old. At that time, her parents entrusted her brother (who was then 4 years old) to her while they were away to work in daytime. This was a common practice in traditional Chinese rural society. One day, the boy fell from a ladder and died. After that, Mary was believed to bring misfortune to her family and she was labeled by her kin as comets (comets symbolize an omen of death and misfortune in Chinese culture. In traditional Chinese community, if bad things happen, women of the family are often labeled as comets, meaning that they are the origin of the bad luck.) afterwards. Because of the stigma, she had encountered numerous situations that made her feel inferior and humiliated (e.g., not allowed to attend her father's funeral). In the interview, Mary could not recall a coherent narrative of this tragedy. She could only access rudimentary memories, including images of a wooden ladder, an old village house, a frightened girl hiding underneath the bed, a loud “bang” sound, and people's noises.

### 2.3. Assessment

Mary had been assessed on the Trauma Symptom Inventory [13] and Impact of Event Scale (IES; [14]) at different points of time. The Trauma Symptom Inventory (TSI) is a self-report questionnaire with good psychometric properties. It assesses posttraumatic stress symptoms and other trauma-related symptoms (e.g., self and other relations). In responding to the TSI, participants are asked to report how often various trauma symptoms have been experienced during the previous 6 months. Ten subscales are formed from the participants' responses. T scores are used to interpret the severity of the stress symptoms and a T score of 65 or above is considered clinically significant. The psychometric properties of the Chinese TSI were found to be satisfactory [15]. The reliability alphas of the subscales of the Chinese TSI in 57 Chinese adults who had come across critical events were as follows: anxious arousal (AA, *α* = .84), depression (D, *α* = .87), anger/irritability (AI, *α* = .82), intrusive experience (IE, *α* = .88), defensive avoidance (DA, *α* = .87), dissociation (DIS, *α* = .82), sexual concern (SC, *α* = .80), dysfunctional sexual behavior (DSB, *α* = .74), impaired self-reference (ISR, *α* = .83), and tension reduction behavior (TRB, *α* = .70).

The Impact of Event Scale (IES; [14]) is another well validated self-report questionnaire designed to assess intrusive and avoidant symptoms in the respondents. A global score (intrusion plus avoidance scores) of 19 is considered to have high and severe impact, while a score below 9 is regarded as having low impact [16]. An unpublished local study on 323 Chinese adults who had encountered traumatic events in their life indicated that the Chinese IES possessed good psychometric properties [15]. Reliability alphas for the intrusion and avoidance subscales and Full scales for the Chinese IES were  .92, .81 and  .90, respectively.

### 2.4. EMDR Treatment

In relation to the target memory of the death of her brother, Mary's NC was “I am responsible for his death.” Given she had difficulty to formulate a PC, she was presented with a list of PC to choose, but the list did not fit her. She later came up with a PC “I can let go of my burden” with a Validity of Cognition Scale (VOC; [11]) of 3. The VOC is a 7-point Likert-type scale where 1 represents an adaptive cognition that is completely unbelievable and a 7 represents one that is totally believable. After several sessions of reprocessing, the VOC remained unchanged. We had a discussion to link up her narrative story line by placing everything came up from the bilateral stimulations (BLS) into perspective. In the course of the eye movements, she began to realize that her brother might be destined to die at an early age, just like it was written in fate that she had to face many hardships in life. This realization was further supported by a visualization of her brother smiling at her during the eye movements in one of the sessions. She interpreted the brother's smile as a kind of love and accepting without blaming. I discussed with her whether the originally specified PC still fit or something better could be used. She felt another PC “I just follow my destined path of life” more appropriate. The VOC of this new PC increased from 4 to 7 after several sessions of reprocessing. At the end of reprocessing, she came up with a thought that “accidents were inevitable when children are left at home unattended.” This reflected that she had accepted the death of her brother as a kind of accident resulting from the negligence of caretakers.

The level of disturbance was measured by using the Subjective Unit of Distress (SUD) scale, where 10 is the highest level of disturbance and 0 is no disturbance. In general, Mary rated 8 to 9 on the SUD scale prior to reprocessing. The bodily sensations (e.g., chest pain, breathing difficulty, stiff shoulder, and hand tremor) associated with the disturbance were also noted. She had nose bleeding intermittently during the initial sessions of reprocessing. With the continuation of eye movements, the bleeding stopped itself. After reprocessing, the SUD dropped to 0 and near to 0. We concentrated on another traumatic event on her abortion experience after the primary event was fully processed. This took only 4 sessions to achieve a resolution. Her symptoms subsided after reprocessing these two events and she no longer met the criteria for PTSD. Given this, we decided to stop using EMDR to reprocess other memories.

**Figure 1:**
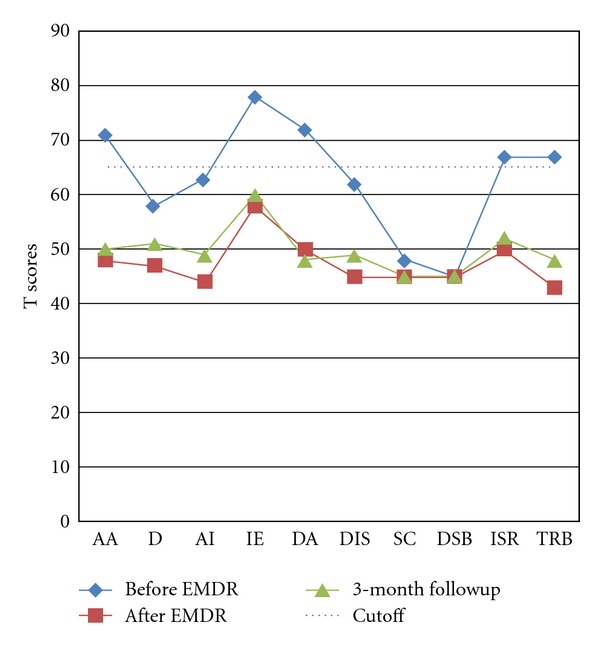
Results of Trauma Symptom Inventory [13] in pretreatment, posttreatment, and three-month followup. *Note*: (1) Trauma Symptom Inventory subscales include anxious arousal (AA), depression (D), anger/irritability (AI), intrusive experience (IE), defensive avoidance (DA), dissociation (DIS), sexual concern (SC), dysfunctional sexual behavior (DSB), impaired self-reference (ISR), and tension reduction behavior (TRB). (2) All symptom scores dropped below clinical level (i.e., T score less than 65) in posttreatment and 3-month followup.

**Figure 2:**
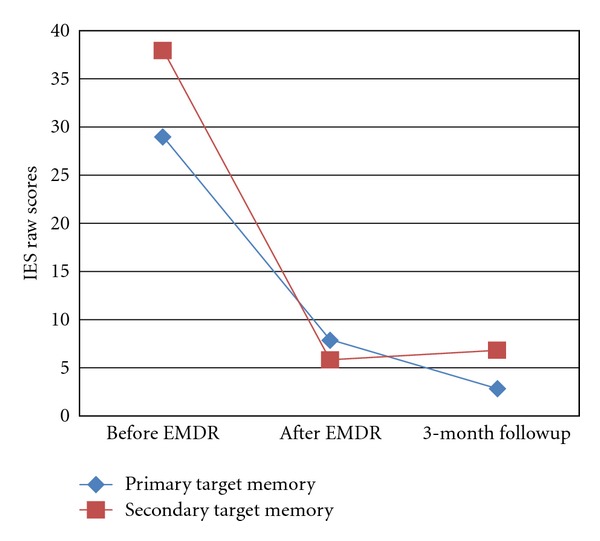
Scores of Impact of Event Scale [14] in pretreatment, posttreatment, and three-month followup. *Note*: the scores of the two target memories dropped below 9 (i.e., having low impact) in post-EMDR and 3-month followup sessions.

In sum, Mary received 21 sessions of EMDR (17 on the death of brother, and 4 on abortion) within a total of 65 therapy sessions over 4 years. Prior to reprocessing, her subscale scores on the Trauma Symptom Inventory (TSI; [13]) ranged from 45 to 78. After reprocessing, all the subscale scores fell below clinical level (see Figure 1). The scores of the Impact Event Scale (IES; [14]) were 29 and 38 (interpreted as having moderate to severe impacts) for her primary and secondary traumatic memories pre-processing, and dropped to 8 and 6 respectively after before exposure (see Figure 2). The decrease in the scores on the TSI and IES were consistent with the change in the ratings of the SUD (from 8/9 10 to 0) and VOC (from 4 to 7). These treatment gains were maintained at a 3-month followup.

At the time of this writing, Mary was receiving training on self-assertion skills. She was also helped to make connection between the past events and her present experience. Bilateral eye movements were sometimes used to strengthen and reinforce the insights and discoveries surfaced during this period.

## 3. Discussion

The case of Mary illustrates the importance of targeting at etiological events in EMDR. The death of Mary's brother lays the foundation of her subsequent pathological responses to other stressful life situations. Apart from the fear engendered from this childhood event, Mary also experienced stigmatization and humiliation imposed by her kin throughout her childhood. These dysfunctional materials were locked in her memory network, and were activated by stressors in life. After reprocessing these earliest memories into an adaptive resolution, positive changes in her affect, cognitions, bodily symptoms, and behaviour were noted. These therapeutic effects were able to generalize to other traumatic events with a similar theme (i.e., loss via death or separation). This is evidenced by the 17 sessions of reprocessing the touchstone event, a significantly less number of sessions (4 sessions) to reprocess the second unpleasant memories, and no reprocessing for other traumatic events. Nonetheless, if Mary has experienced other traumatic events of dissimilar nature, each of these events have to be targeted separately [11].

This case also illustrates the importance of identifying a PC that fits client's culture. Mary initially could not think of an appropriate PC. She also found the list of PC offered to her not suitable. She randomly thought of a PC “I can let go of my burden.” This PC seemingly did not fit her. The subsequent emerged statement “I just follow my destined path of life” may sound passive and somewhat resigning, but it turned out more suitable to her.

To the Chinese people, accepting one's fate and destiny is a kind of active taking of the bad situations. It is similar to a Chinese idiom “let nature take its course.” To Mary, this cognition also implies that she should not take sole responsibility for her brother's death. Obviously, there is some PC which is universal and comprehensible to both Western and Chinese people (e.g., “I am safe,” “I am useful/worthwhile/valuable”). However, some cognition may mean negative to Western people but not to Chinese people. To Chinese, the statement “I just follow the fate” means receiving and accepting what is offered by the nature. It has a connotation of “I have done my best but nature has its way.” Mary's case illustrates that accurate matching of a PC appropriate to one's cultural belief would allow the capturing of the core theme of traumas, and provides a better chance for successful treatment.

## References

[1] Van Der Kolk BA, Pelcovitz D, Roth S, Mandel FS, McFarlane A, Herman JL (1996). Dissociation, somatization, and affect dysregulation: the complexity of adaptation to trauma. *American Journal of Psychiatry*.

[2] Courtois CA (2004). Complex trauma, complex reactions: Assessment and treatment. *Psychotherapy*.

[3] Curtois C (2000). Complex trauma, complex reactions: assessment and treatment. *Psychological Trauma: Theory, Research, Practice, and Policy*.

[4] Van Der Kolk BA, Roth S, Pelcovitz D, Sunday S, Spinazzola J (2005). Disorders of extreme stress: the empirical foundation of a complex adaptation to trauma. *Journal of Traumatic Stress*.

[5] Cardena E, Maldonado J, Van der Hart O, Spiegel. D, Foa EB, Keane TM, Friedman MJ (2000). Hypnosis. *Effective Treatments for PTSD*.

[6] Ford JD, Courtois CA, Steele K, Van Der Hart O, Nijenhuis ERS (2005). Treatment of complex posttraumatic self-dysregulation. *Journal of Traumatic Stress*.

[7] Poon MWL (2009). Hypnosis for complex trauma survivors: four case studies. *American Journal of Clinical Hypnosis*.

[8] Bradley R, Greene J, Russ E, Dutra L, Westen D (2005). A multidimensional meta-analysis of psychotherapy for PTSD. *American Journal of Psychiatry*.

[9] Bisson JI, Ehlers A, Matthews R, Pilling S, Richards D, Turner S (2007). Psychological treatments for chronic post-traumatic stress disorder: systematic review and meta-analysis. *British Journal of Psychiatry*.

[10] Davidson PR, Parker KCH (2001). Eye movement desensitization and reprocessing (emdr): a meta-analysis. *Journal of Consulting and Clinical Psychology*.

[11] Shapiro F (2001). *Eye Movements Desensitization and Reprocessing: Basic Principles, Protocols and Procedures*.

[12] Shapiro F, Shapiro F (2002). Paradigms, processing, and personality development. *EMDR as an Integrative Psychotherapy Approach: Experts of Diverse Orientations Explore the Paradigm Prism*.

[13] Briere J (1995). *Trauma Symptom Inventory: Professional Manual*.

[14] Horowitz M, Wilner N, Alvarez W (1979). Impact of event scale: A measure of subjective stress. *Psychosomatic Medicine*.

[15] Liaison Group on Test Management (2005). *Findings of Impact of Event Scale study using Hong Kong samples*.

[16] Horowitz MJ (2000). *Personal Communication with Liaison Group on Test Management of Social Welfare Department of Hong Kong*.

